# Magnetic Properties and Large Second-Harmonic Generation Response of a Chiral Ternary Chalcogenide: Eu_2_SiSe_4_

**DOI:** 10.1021/acs.chemmater.5c00421

**Published:** 2025-07-09

**Authors:** Shaun O’Donnell, Ian A. Leahy, Subhendu Jana, Eric A. Gabilondo, P. Shiv Halasyamani, Paul A. Maggard, Rebecca W. Smaha

**Affiliations:** † Materials Science Center, 53405 National Renewable Energy Laboratory, Golden, Colorado 80401, United States; ‡ Department of Chemistry and Biochemistry, 14643 Baylor University, Waco, Texas 76798, United States; § Department of Chemistry, 14743 University of Houston, Houston, Texas 77204, United States; ∥ Materials Science Center, National Renewable Energy Laboratory, Golden, Colorado 80401, United States

## Abstract

Eu­(II)-containing chalcogenides are an emerging class of materials that are of great interest due to their high optical activity and intriguing magnetism. Here, we synthesized Eu_2_SiSe_4_ as red-colored single crystals and characterized its structure with single-crystal X-ray diffraction, confirming the reported chiral monoclinic *P*2_1_ symmetry at room temperature. The crystal structure of Eu_2_SiSe_4_ comprises distorted SiSe_4_ tetrahedral units and charge-balancing Eu­(II) cations. Here, we develop a two-step solid-state synthesis method for Eu_2_SiSe_4_ and compare it to the known boron chalcogenide method. We find the second-harmonic generation (SHG) activity of polycrystalline Eu_2_SiSe_4_ to be ∼7 × AgGaS_2_, placing it among the highest-known SHG-active chalcogenides. No symmetry lowering is observed down to 100 K in single-crystal X-ray diffraction, although an anomalous expansion in the *b*-axis lattice parameter occurs and may be correlated to lattice modes of the SiSe_4_ tetrahedra. We investigate the physical properties of Eu_2_SiSe_4_ using magnetometry and heat capacity measurements and find a transition to an antiferromagnetic ground state at *T*_*N*_ ≈ 5.5 K. The low-temperature transition releases less entropy than expected, which may be due to the complex crystal electric field effects of Eu­(II).

## Graphical abstract

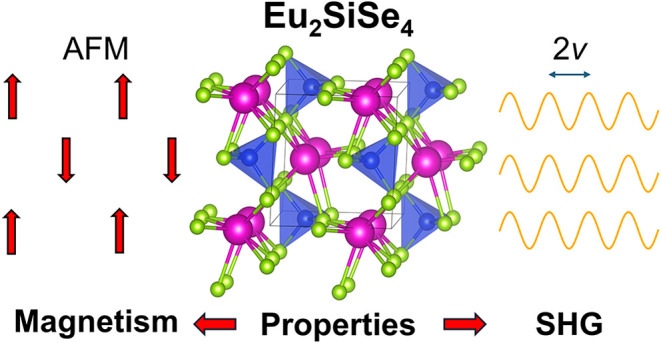

## Introduction

Solid-state chemistry plays a vital role in discovering the compounds which go on to form the materials and technologies that enable our quality of life. However, solid-state chemistry is not just a synthesis pursuit; it also aims to understand the underlying relationships between a compound’s structure and its properties to develop a deeper understanding of the origin of their useful properties. In turn, these insights enable scientists and engineers to discover and fabricate better functional materials. As such, fundamental studies on new or underexplored compounds form the bedrock of our modern technologies.

One such family of compounds showing promise is the metal chalcogenides. Metal chalcogenides are finding uses in fields such as photovoltaics,^1,2^ nonlinear optics,^3–5^ superconductivity,^6,7^ thermoelectrics,^8–10^ and topological materials.^11,12^ Moreover, chalcogenides often tend to adopt broader, and more complex, crystalline structures compared to oxides. This offers the possibility for greater structural and compositional tuning in chalcogenides, and thus, greater capacity for tuning properties. To this end, we have recently been exploring europium-containing chalcogenides. Europium is somewhat unique among the rare-earth elements in that it can adopt a 2+ valence state in addition to the typical 3+ valence found in most other rare-earths. Europium’s 2+ valence state is also interesting in that it results in a 4*f* ^7^ (*S* = 7/2) electronic configuration, which can lead to intriguing magnetic properties.^13^ Additionally, we have also reported several new Eu-containing chalcogenides with large second-harmonic generation (SHG) in the mid-infrared region.^14^ These results have therefore motivated us to further explore this emerging phase space.

Recently, Panigrahi et al. independently reported the discovery of Eu_2_SiSe_4_ via the boron chalcogenide mixture (BCM) method of synthesis.^15^ They found Eu_2_SiSe_4_ adopts a chiral crystal structure, *P2*_1_, at room temperature. The chirality offers the possibility for intriguing optical and magnetic properties; however, property measurements beyond ultraviolet–visible (UV–vis) diffuse reflectance measurements to obtain the optical bandgap have yet to be reported. Here, we expand on the initial characterization of Eu_2_SiSe_4_ and further elucidate its crystalline structure and properties. We successfully synthesized Eu_2_SiSe_4_ as single crystals and bulk crystalline powder using both the BCM technique and a two-step solid-state synthesis method. In addition to confirming the room temperature crystal structure, we also performed single-crystal X-ray diffraction at 100 K and found a subtle, and unusual, structural change involving increasing Eu–Se bond distances at low temperature. The chiral nature of the compound was further validated via SHG, where the SHG response is one of the largest reported to date. Finally, the magnetic and thermodynamic properties were characterized for the first time, revealing a transition to an antiferromagnetic ground state at *T*_*N*_ ≈ 5.5 K. We discuss how the synthesis method influences the presence of impurities (namely EuSe) and complicates measurement of the intrinsic properties of Eu_2_SiSe_4_.

## Experimental Section

### Synthesis

Eu_2_SiSe_4_ was synthesized as single crystals and in bulk polycrystalline form starting from elemental starting materials of Eu (chunk, Alfa Aesar, 99.9% purity), Eu_2_O_3_ (powder, Alfa Aesar, 99.9% purity), B (powder, BTC, 99.99% purity), Si (powder, Alfa Aesar, 99.99% purity), and Se (powder, Alfa Aesar, 99.999% purity). As Eu is air and moisture-sensitive, all chemical manipulations were carried out inside an Ar-filled glovebox.

#### Growth of Eu_2_SiSe_4_ Crystals

Red block-shaped crystals of Eu_2_SiSe_4_ were synthesized via the solid-state method using carbon-coated fused silica ampules with 4 mm inner diameter (ID) and 6 mm outer diameter (OD) as reaction vessels. Stoichiometric amounts of Eu chunks (93.8 mg, 0.62 mmol), Si powder (8.7 mg, 0.31 mmol), and Se powder (97.5 mg, 1.23 mmol) were loaded into the ampule. The tube was then flame-sealed under 10^–4^ Torr and heated to 1223 K in a programmable muffle furnace in 16 h and soaked for 48 h before switching off the furnace and allowing it to radiatively cool to room temperature. The ingot product was crushed and analyzed in ambient conditions under an optical microscope, revealing red block-shaped crystals. Selected crystals were analyzed using energy dispersive X-ray (EDX) spectroscopy in a JEOL SEM 6010LA; samples were mounted on carbon tape.

#### Synthesis of Polycrystalline Eu_2_SiSe_4_ via a Two-Step Solid-State Method

A polycrystalline sample of Eu_2_SiSe_4_ was synthesized using a two-step solid-state synthesis method. In the first step, stoichiometric amounts of Eu chunks (281.5 mg, 1.85 mmol), Si powder (26 mg, 0.93 mmol), and Se powder (292.5 mg, 3.70 mmol) were loaded into a 10 mm ID/12 mm OD fused silica tube and flame-sealed under dynamic vacuum of 10^–4^ Torr. The tube was then heated to 1223 K over 16 h and annealed for 60 h before switching off the furnace. The product was ground inside the glovebox, pelletized, and then sealed inside a fused silica tube under dynamic vacuum of 10^–4^ Torr. The sealed ampule was heated at 973 K for 72 h before switching off the furnace. The product was ground and homogenized inside the glovebox.

#### Synthesis of Polycrystalline Eu_2_SiSe_4_ via a Two-Step Solid-State Method

The Eu_2_SiSe_4_ phase was also synthesized using the boron chalcogen mixture (BCM) method. Stoichiometric amounts of Eu_2_O_3_ powder (490.5 mg, 1.39 mmol), Si powder (39.1 mg, 1.39 mmol), Se powder (440.2 mg, 5.58 mmol), and B powder (30.1 mg, 2.79 mmol) were loaded into a 12 mm OD/10 mm ID heavily carbon-coated fused silica tube in ambient atmosphere and flame-sealed under dynamic vacuum of 10^–4^ Torr. The sealed ampule was then ramped to 1223 K over 14 h and annealed for 48 h before being cooled to 773 K using a cooling rate of 20 K/h. Finally, the furnace was switched off and allowed to cool down to room temperature. The tube was cracked open inside a fume hood, and the product was washed with ethanol to remove boron oxides and allowed to dry.

### Characterization Using X-ray Diffraction Techniques

The crystal structure of the ternary chalcogenide Eu_2_SiSe_4_ was established using single-crystal X-ray diffraction (SCXRD) at 300(2) K and 100(2) K using Bruker D8 Venture and a Bruker D8 Quest diffractometers, respectively. A block-shaped, red-colored single crystal of Eu_2_SiSe_4_ was measured using the diffractometers equipped with a Photon III mixed-mode detector and a monochromatized Mo–Kα radiation source. A suitable SEM-EDX-analyzed Eu_2_SiSe_4_ single crystal was mounted on a Kapton loop under viscous Paratone-N. The APEX4 software^16^ was used to collect and reduce the data. The crystal quality and unit cell were initially judged from 180 frames of fast scan data. For the full data collection, a frame width, crystal-to-detector distance, and exposure time of 0.5°, 50 mm, and 3 s/frame, respectively, were used. Finally, the data were integrated and the absorption correction was carried out using the multiscan method in SADABS.^17^

XPREP^18^ suggested the chiral space group *P*2_1_ based on the average |*E* ^2^ – 1 | (intensity statistics) value and extinction conditions. The structures were solved using SHELXT,^19^ and the anisotropic displacement parameters, atomic positions, scale factors, weight corrections, and extinction corrections were refined using the SHELXL least-squares method.^20^ The crystals were twinned; we employed the inversion twin law [-1 0 0 0 -1 0 0 0 -1]. PLATON ADDSYM^21^ did not suggest any additional symmetry, validating the structural model. The STRUCTURE TIDY program^22^ was finally used to standardize the atomic positions of the Eu_2_SiSe_4_ crystal structure. The refinement and structural details are provided in Tables S1–S4. Bond valence sum calculations were performed using EXPO2013.^23^

The phase purity of polycrystalline Eu_2_SiSe_4_ samples were studied using room temperature powder X-ray diffraction (PXRD) using a PANalytical Empyrean X-ray diffractometer and a Bruker D2 Phaser diffractometer for the solid-state and BCM samples, respectively; both with Cu–Kα radiation sources. Rietveld refinements were performed using GSAS-II.^24^

### Optical Spectroscopy and Second-Harmonic Generation

Mid-infrared (IR) second-harmonic generation (SHG) data were recorded using a Ho: YAG laser. The sample was measured at room temperature using a wavelength of 2.09 μm and a modified Kurtz-Perry system.^25^ The polycrystalline Eu_2_SiSe_4_ sample was finely ground to record the frequency-doubled output data using a photomultiplier tube. A polycrystalline AgGaS_2_ (AGS) sample was used as the standard reference for the SHG measurement.

The optical bandgap of the polycrystalline Eu_2_SiSe_4_ sample synthesized via the solid-state method was obtained from diffuse reflectance data using a Shimadzu UV3600 spectroscopy instrument. The sample was measured as a function of wavelength from 1000 nm (1.24 eV) to 250 nm (4.96 eV) using dried BaSO_4_ as a standard reference. The Kubelka–Munk equation (α/*S* = (1 – *R* ^2^) /(2*R*)) was used to transform the reflectance data to absorption data. Here *S*, *R*, and α are scattering coefficients, reflectance, and absorption coefficients, respectively.^26^ A Tauc plot^27^ was used to obtain the optical bandgap of Eu_2_SiSe_4_:(α*hγ*)^*n*^ = *A*(*hγ* – *E*_g_). Here *E*_g_, *A*, *h*, and γ are bandgap, proportionality constant, Planck’s constant, and frequency of light, respectively. The *n* = 2 and $\frac{1}{2}$ values represent the direct and indirect bandgaps, respectively.

### Physical Property Measurements

Magnetic susceptibility and DC magnetization measurements were performed in a Quantum Design Physical Property Measurement System (PPMS) in the range 2–400 K in fields up to μ_0_ *H* = 14 T. Heat capacity was measured in the PPMS up to 200 K and 14 T. Apiezon N-grease was used to adhere a small chunk of pelletized powder to the puck. To analyze the magnetic contribution to the heat capacity around the magnetic ordering temperature, a background heat capacity (*C*_BG_) must be subtracted. We fit a Debye model with a two-level Schottky term above 30 K to generate this background. Raw heat capacity data are interpolated for the calculation of *S*_Mag_.

## Results and Discussion

### Synthesis and Crystal Structure

Recently, Panigrahi et al. reported the synthesis of single crystals of Eu_2_SiSe_4_ using the boron chalcogenide mixture (BCM) method, which utilizes oxides as starting materials and boron as a scavenger of oxygen from the oxide starting materials.^15^ Here, single crystals and powders of red-colored ternary chalcogenide Eu_2_SiSe_4_ were synthesized from direct synthesis of elemental starting materials using a solid-state (SS) synthesis method at 1223 K (see Experimental Section for details). The composition of the crystals was investigated using energy dispersive X-ray spectroscopy (EDX) in a scanning electron microscope, showing the presence of Eu, Si, and Se in the ratio of 13.85:28.01:58.14 (∼2:1:4).

The structure of Eu_2_SiSe_4_ crystals grown via the SS method was determined at 100 and 300 K using single-crystal X-ray diffraction (SCXRD) data (Figure 1 and Tables S1–S4), confirming that Eu_2_SiSe_4_ crystallizes in the monoclinic, chiral *P*2_1_ space group at room temperature and maintains this symmetry down to 100 K. The structure consists of distorted, pseudo zero-dimensional [Si_1_Se_4_]^4–^ tetrahedral units and Eu_1_Se_7_ and Eu_2_Se_8_ polyhedral units, and the Eu atoms form a highly distorted cubic lattice.

**Figure 1:**
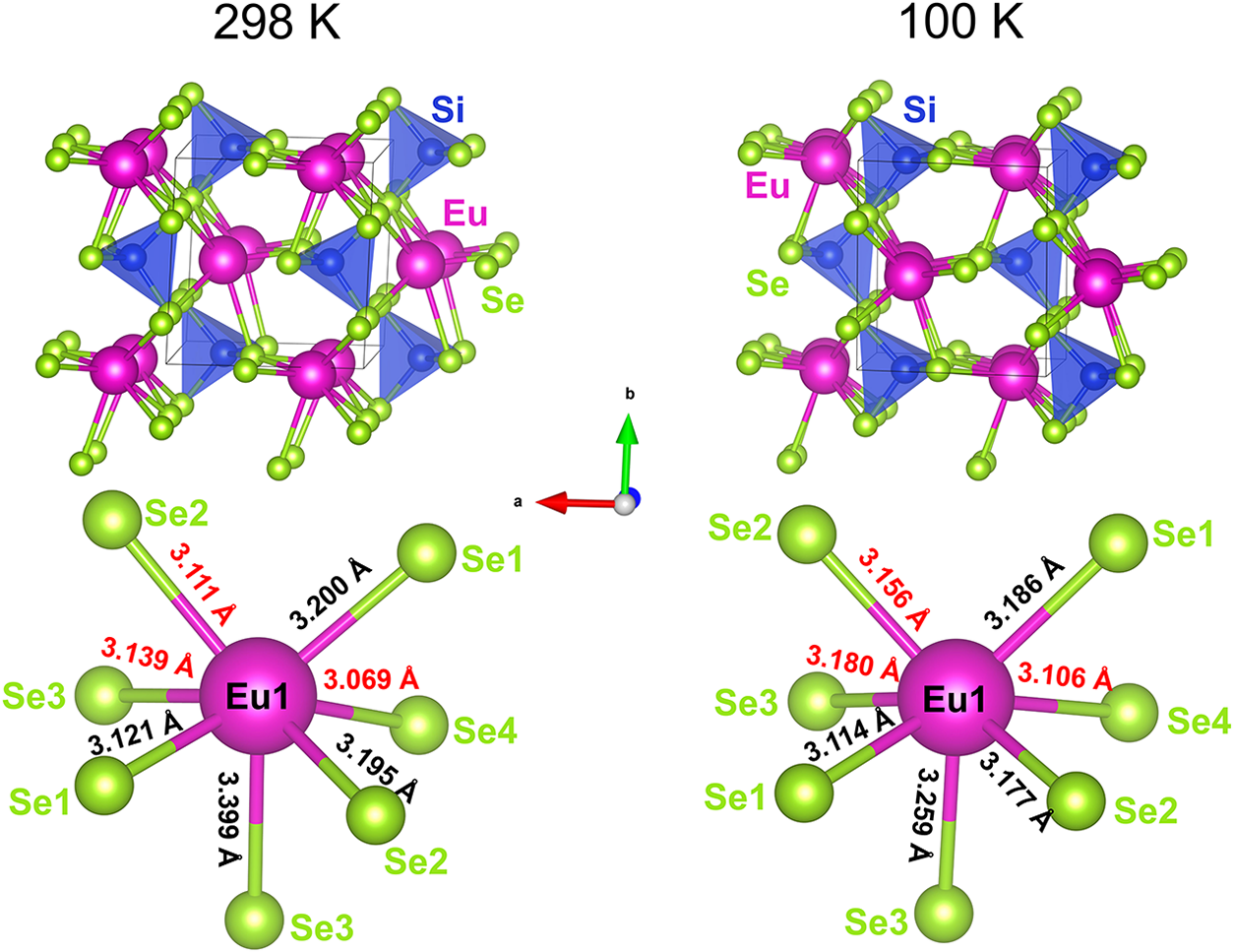
Depiction of the Eu_2_SiSe_4_ crystal structure at room temperature and 100 K. The symmetry and overall structure of Eu_2_SiSe_4_ is similar at both temperatures, but shows unexpected lengthening of some Eu–Se bonds at the lower temperature (highlighted in red).

The S analog of Eu_2_SiSe_4_ (Eu_2_SiS_4_) and the S-substituted variants (from Eu_2_SiSe_0.85_S_3.15_ to Eu_2_SiSe_3.1_S_0.9_) were reported to crystallize in the centrosymmetric space group *P*2_1_/*m* at room temperature.^15^ As discussed further below, the SHG activity of our polycrystalline Eu_2_SiSe_4_ samples confirms the chiral nature of the Eu_2_SiSe_4_ crystal structure, establishing the *P*2_1_ space group for Eu_2_SiSe_4_ down to at least 100 K.

The structural comparison between the crystal data collected at 300 and 100 K for Eu_2_SiSe_4_ suggests that the material maintains its monoclinic, chiral *P*2_1_ symmetry across the two temperatures, with small but interesting changes in the overall unit cell dimensions. The decrease in unit cell volume (∼0.86%, or ∼2.83 Å^3^) from 300 to 100 K is expected as the crystal contracts upon cooling. Interestingly, while the *a*- and *c*-axes both contract (∼0.5 to 0.6%), the *b*-axis unexpectedly expands (∼0.2%) at lower temperatures.

The most significant changes in interatomic distances occur for the Eu1–Se3 and Eu2–Se4 bond lengths, with both distances slightly shrinking. Specifically, the Eu1–Se3 distance decreases by ∼3%, from 3.3992(8) Å at 300 K to 3.297(1) Å at 100 K. The Eu2–Se4 bond length also shortens by ∼3%, from 3.367(1) Å at 300 K to 3.259(1) Å at 100 K. Most interestingly, both interatomic distances are principally aligned down the *b*-axis, which is the only axis that undergoes thermal expansion at lower temperatures. These contrasting changes may arise from “rigid unit mode” type of lattice vibrations of the SiSe_4_ tetrahedra, as the Si–Se distances remain relatively unchanged, and causing small but detectable changes in the Eu–Se coordination environments.

As Eu_2_SiSe_4_ is a red-colored semiconducting (see below) compound, charge balancing can be carried out using the Zintl–Klemm concept considering the elements at their most stable closed-shell electronic configuration of the elements.^29^ The absence of short homoatomic bonding distances confirms the lack of polyselenide bonding in the Eu_2_SiSe_4_ crystal structure, indicating the +4 and −2 oxidation states of Si and Se atoms. While the Eu could be in either +2 or +3, charge balancing suggests +2. BVS calculations for Eu_2_SiSe_4_, tabulated in Tables S2 and S3, agree with the +2, +4, and −2 oxidation states for Eu, Si, and Se atoms, respectively.

The phase purity of polycrystalline Eu_2_SiSe_4_ synthesized via the SS method was investigated using a Rietveld refinement of room temperature powder X-ray diffraction (PXRD) data, revealing an EuSe impurity phase at 14.7(2) wt %. For comparison, we also synthesized polycrystalline Eu_2_SiSe_4_ using the BCM method (see Experimental Section for details); a Rietveld refinement of PXRD data of this sample revealed much less EuSe present at 2.5(2) wt %. The PXRD data for the SS and BCM samples are compared in Figures S1 and S2.

### Optical Properties

Recently, Eu-containing chalcogenides have garnered interest because of their high SHG activity.^30^ We studied the SHG response of polycrystalline samples of Eu_2_SiSe_4_ synthesized by both SS and BCM methods at a wavelength of 2.09 μm using a Ho: YAG laser. Both samples were found to be SHG active (Figure 2), which confirms the chiral symmetry of Eu_2_SiSe_4_ (space group *P*2_1_) at room temperature. The SHG activity of the SS polycrystalline Eu_2_SiSe_4_ sample was found to be approximately seven times than that of AGS (AgGaS_2_) and one of the largest reported to date. As shown in Figure 2, the data were measured twice to check the consistency of the SHG signal and were found to be consistent. A comparison of the SHG activity of Eu_2_SiSe_4_ is provided in Table 1. However, the BCM Eu_2_SiSe_4_ sample showed a SHG activity close to that of AGS, even though it had approximately the same amount of the EuSe impurity (which should not contribute to the measurements due to its cubic rocksalt structure) as the SS sample. We synthesized and measured a second SS sample, but it yielded SHG activity of approximately 0.4 times AGS (see Figure S3a). Phase matching experiments were performed on the BCM and second SS samples (Figure S3b). We note that the discrepancies between the measurements may be due to the optical quality or size of the crystallites and will be left for future research (and researchers) to investigate further.

**Figure 2:**
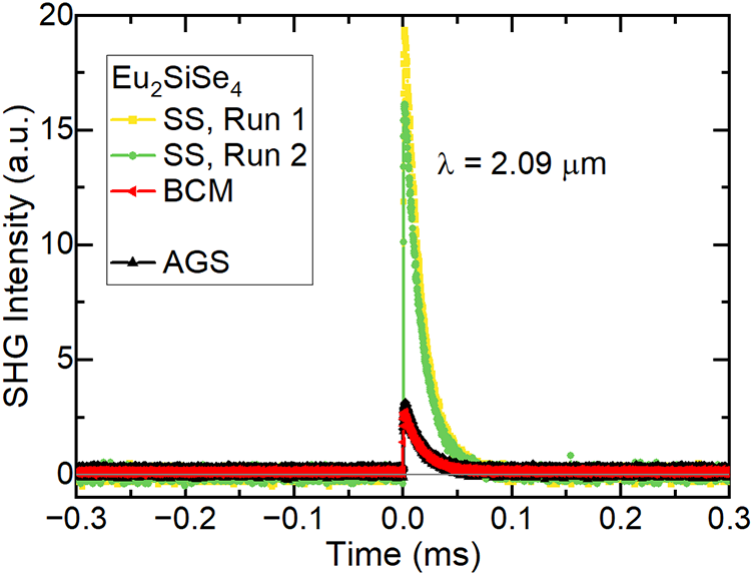
SHG response at 2.09 μm of Eu_2_SiSe_4_ against AGS (AgGaS_2_), comparing the solid-state (SS) and BCM synthesis methods. The SS sample was measured twice to check the consistency. The particle sizes of the SS, BCM, and AGS samples were ∼1–50, 63–75, and 63–75 μm, respectively.

**Table 1:** Comparison of High SHG Responses in Eu_2_SiSe_4_ and Previously Reported Chalcogenides

compound	space group	bandgap (eV)	SHG × AGS	reference
AgGaS_2_	*I*4̅2*d*	2.64	1	^31^
Eu_2_SiSe_4_	*P*2_1_	1.9	∼7.0	this work
Eu_3_Ag_2_Sn_2_S_8_	*I*4̅3*d*	2.0	∼7.0	^14^
Ba_3_CdSn_2_S_8_	*I*4̅3*d*	2.30	∼0.8	^32^
Sr_3_Ag_2_Ge_2_S_8_	*I*4̅3*d*	2.62	∼1.3	^33^
EuCdGeSe_4_	*Ama*2	2.25	∼3.8	^34^
Hg_3_Na_2_Ge_2_S_8_	*I*4̅3*d*	2.68	∼3.0	^35^

Optical absorption data of polycrystalline Eu_2_SiSe_4_ synthesized via the SS method were collected at room temperature in diffuse reflectance mode, and the bandgap was estimated using the Tauc method (Figure 3). The direct and indirect bandgaps were found to be 1.93(2) eV and 1.87(2) eV, respectively. As the difference between the direct and indirect bandgaps is less than 0.1 eV, the bandgap of polycrystalline Eu_2_SiSe_4_ sample is pseudodirect. The red color of the compound and observed bandgap also agrees with the bandgap determined by the Tauc method and is similar to the previously reported bandgap of 1.90(2) eV for polycrystalline Eu_2_SiSe_4_ synthesized via the BCM method.^15^

**Figure 3:**
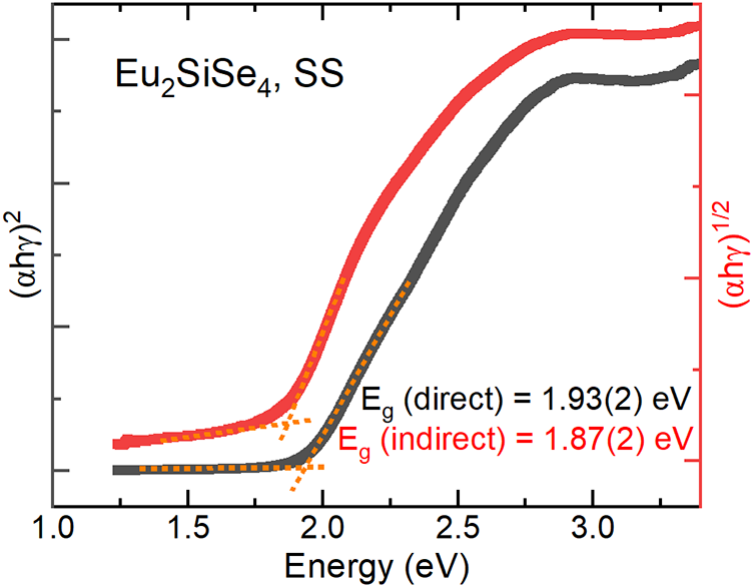
Tauc plot of polycrystalline Eu_2_SiSe_4_ synthesized via the SS method measured at room temperature in diffuse reflectance mode.

### Magnetic Properties

Magnetic susceptibility was measured as a function of temperature under a variety of applied magnetic fields, as shown in Figure 4. A clear cusp is observed at ∼5.5 K in the lower-field data, *μ*_0_ *H* ≤ 1 T, and some splitting between zero-field-cooled and field-cooled data is observed at *μ*_0_ *H* = 0.005 T (Figure S5). This behavior is consistent with an antiferromagnetic-like ordering transition at approximately *T*_N_ ≈ 5.5 K. A small peak is also observed at ∼4.7 K; this most likely arises from the small (2.5(2) wt.%) EuSe impurity in this sample.^36–38^ This assignment is confirmed by the increase in intensity of this 4.7 K peak observed in data collected on the SS sample, which contains 14.7(2) wt.% EuSe, as shown in Figure S4. At intermediate fields, the cusp broadens, and the data are nearly linear at high fields.

**Figure 4:**
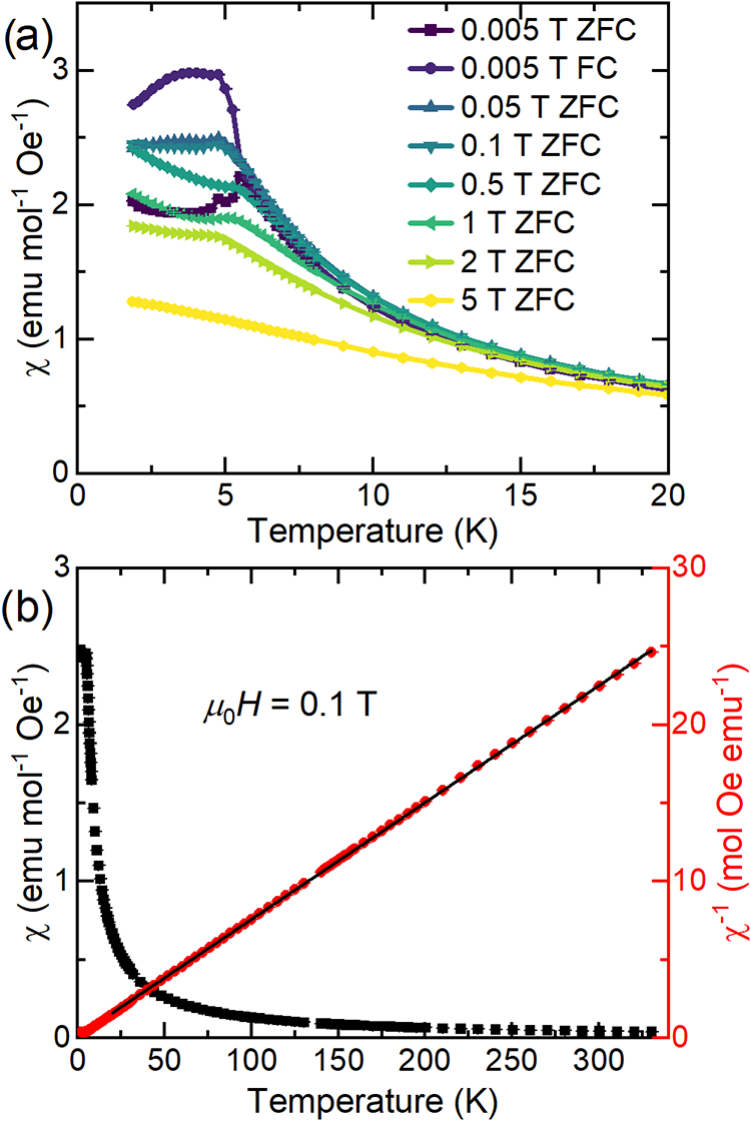
(a) Low-temperature magnetic susceptibility (χ) of polycrystalline Eu_2_SiSe_4_ synthesized via the BCM method as a function of temperature measured at several applied fields. (b) Susceptibility and inverse susceptibility (χ^–1^) measured in an applied field of *μ*_0_ *H* = 1 T. The Curie–Weiss fit is the black line. All data are zero-field cooled (ZFC) unless otherwise stated in the legend.

We measured to high temperature to perform a Curie–Weiss fit from 20–350 K with no diamagnetic correction χ_0_, as shown in Figure 4b. This yielded a Weiss temperature Θ = −1.6(2) K, which is consistent with weak antiferromagnetic interactions and the low observed *T*_N_ ≈ 5.5 K. The extracted Curie constant is *C* = 13.41(1) K·emu/mol per formula unit, and we calculate an effective moment of 7.323(4) μ_B_ per Eu, which is lower than the expected value of 8 μ_B_, suggesting that approximately 80% of the sample is in the 2+ oxidation state suggested by BVS calculations. The presence of approximately ∼20% Eu­(III) likely occurred during the washing step necessary to remove boron oxides. The SS synthesis method does not contain this step, and the effective moment that we extracted from a Curie–Weiss fit of this data is 8.1(1) μ_B_ per Eu, consistent with Eu­(II) (see Figure S4c and the Supporting Information for more details).

Magnetization as a function of applied field was measured at several temperatures, shown in Figure 5a. No hysteresis is observed below the *T*_N_ ≈ 5.5 K transition. The data at *T* = 2 K show several changes in slope at low field, consistent with the susceptibility results; this is more clearly seen in the several peaks present in the derivative of magnetization as a function of applied field (*dM*/*dH*, Figure 5b). This behavior persists through *T* ≈ 4 K and then disappears above the ordering temperature of the small EuSe impurity phase (4.7 K). At *T* = 5 K, at which point the only magnetically-ordered phase should be Eu_2_SiSe_4_, the *dM*/*dH* data show a broad hump at approximately 1.5 T and a sharp upturn near zero field. Both of these features decrease above the antiferromagnetic ordering temperature. By comparing data sets for the SS and BCM samples (see Figure S7), it becomes apparent that the feature at ∼1 T arises from Eu_2_SiSe_4_ while the strong features at ∼3 T and ∼0.5 T likely arise primarily from EuSe. Interestingly, the feature due to Eu_2_SiSe_4_ shifts toward higher applied fields with increasing temperature, while the features due to EuSe shift toward lower applied fields as the temperature increases, indicative of the weakening of the magnetic interactions at higher temperatures.

**Figure 5:**
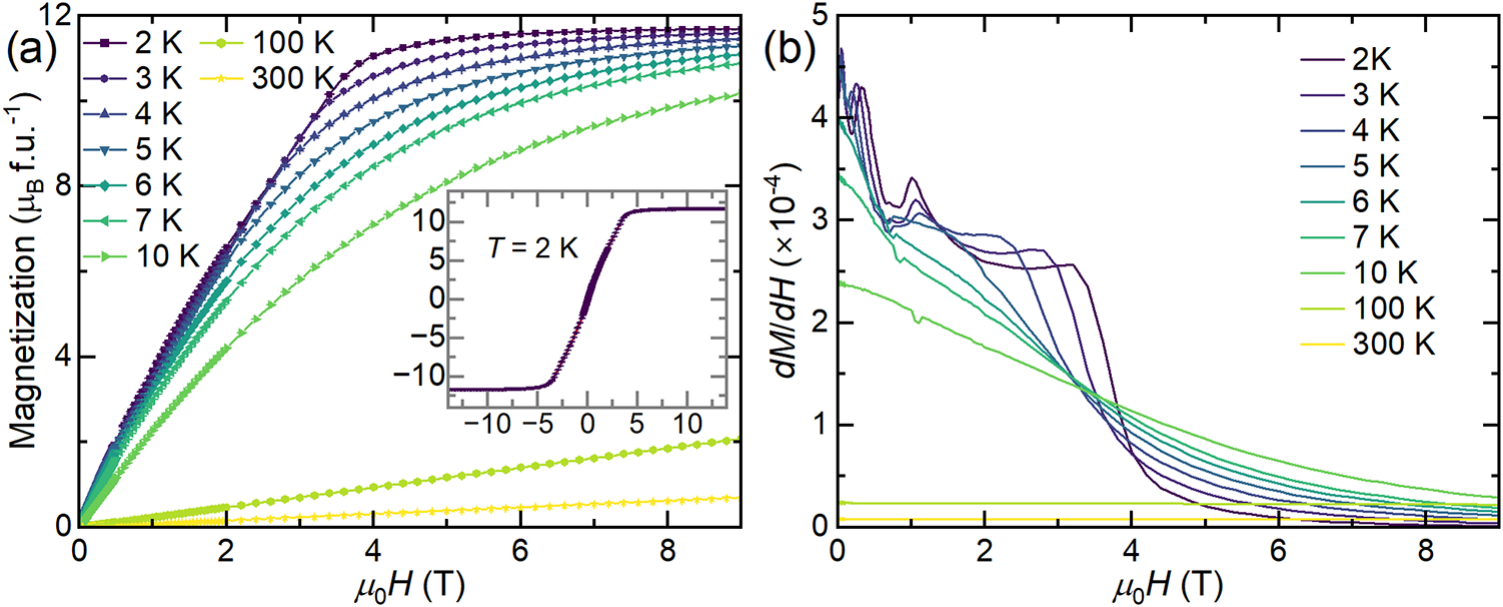
(a) DC magnetization as a function of applied field at several temperatures. Inset shows the full field loop for *T* = 2 K. (b) Derivative of magnetization as a function of applied field at several temperatures.

### Thermodynamic Properties

Figure 6a shows the temperature dependence of the heat capacity from 1.9–30 K at fixed magnetic fields up to 14 T. At 0 T, a sharp lambda peak at ∼5.3 K is clearly visible, consistent with the susceptibility data. With increasing field, this peak shifts to lower temperature, confirming its magnetic origin. Above 1 T, the peak broadens significantly. Heat capacity was also measured on the SS sample, and the two samples are compared in Figure S8. Consistent with the susceptibility and magnetization data, the heat capacity of the SS sample shows a larger contribution from EuSe.

**Figure 6:**
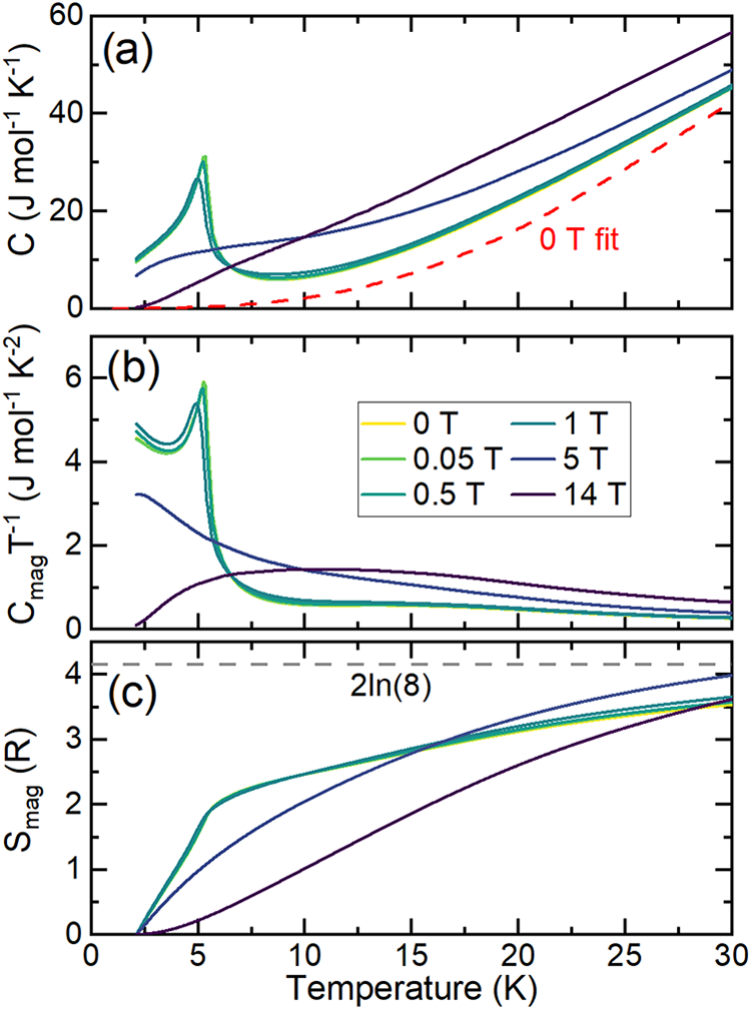
Heat capacity for polycrystalline Eu_2_SiSe_4_ synthesized by the BCM method. (a) Molar heat capacity (C_p_) at several applied fields; red dashed line represents the background (*C*_bg_) for the *μ*_0_ *H* = 0 T curve, which was fit above 30 K to a Debye model with a two-level Schottky contribution and extrapolated to zero. (b) Heat capacity is related to the low temperature peaks (*C*_mag_). (c) Magnetic entropy at low temperature (*S*_mag_) normalized per formula unit.

To analyze the magnetic contributions to the heat capacity, we fit the 0 *T* heat capacity above 30 K to a Debye model with a two-level Schottky contribution to account for thermally activated crystal electric field (CEF) split states (red dashed line in Figure 6a; see also Figure S9). We plot *C*_mag_/*T* in Figure 6b where *C*_mag_ is the magnetic contribution to the heat capacity defined as *C*_mag_(*T,H*) ≡ *C*(*T,H*)– *C*_BG_(*T*) . Figure 6c shows the released entropy determined from an integral of *C*_mag_/*T* for μ_0_ *H* = 0 T. The effective moment of 7.323(4) μ_B_ per Eu extracted from the Curie–Weiss fit indicates *L* = 0 and *S* = 7/2, giving an expected released entropy per Eu atom of *R* log 8 (or 2*R *log 8/mol, dashed line). We anticipate that the disagreement of the experimental *S*_Mag_ originates from a combination of the presence of some oxidized Eu­(III), magnetic fluctuations persisting above the Néel temperature, and CEF effects. This is reflected in the magnetic field dependence of the heat capacity far above *T*_N_ as well as the broadness of this dependence in temperature. It is likely the case that the manifold of CEF states is not faithfully captured by our simple two-level model. In future work, we anticipate that a more complete understanding of energy splittings between the most relevant Eu­(II) CEF states would resolve this discrepancy.

## Conclusions

We synthesized red-colored single crystals and powders of Eu_2_SiSe_4_ using both solid-state and boron chalcogenide mixturemethods. Structural characterization using SCXRD confirmed the polar, chiral *P*2_1_ symmetry previously reported at room temperature. Measuring SCXRD at low temperature for the first time revealed no symmetry lowering down to 100 K, although an anomalous expansion of the *b*-axis is observed. Further confirming the chiral symmetry, we measured SHG activity in Eu_2_SiSe_4_ at room temperature—among the highest reported for chalcogenides to date—although this strong activity was not highly reproducible. Tauc fitting of diffuse reflectance optical absorption data revealed a pseudodirect bandgap at approximately 1.9 eV.

Magnetic measurements confirmed the presence of Eu­(II) and reveal that Eu_2_SiSe_4_ undergoes a transition to an antiferromagnetic ground state at *T*_N_ ≈ 5.5 K, consistent with the small, negative Weiss temperature extracted from the data. Heat capacity data confirm the presence and magnetic field dependence of this transition. We find that the released entropy in the magnetic transitions near *T*_N_ falls short of the expected values, likely stemming from a combination of complex crystal electric field effects, some oxidation of Eu­(II) to Eu­(III), and the persistence of magnetic fluctuations above *T*_N_. As these measurements were complicated by the presence of the EuSe impurity phase in samples synthesized by both methods explored here, care must be taken to ensure phase purity and fully characterize samples of interest. In addition, the future growth of larger single crystals would enable further studies of the intrinsic properties of Eu_2_SiSe_4_, including electronic transport, neutron scattering to illuminate the magnetic structure, and possible anisotropic magnetic behavior, which may be intriguing given the anomalous temperature dependence of the lattice parameters.

## Supplementary Material



## Notes

Crystallographic details of the SCXRD structures; PXRD data; and additional SHG, magnetism, and heat capacity data (PDF)

## References

[1] Hadke S., Huang M., Chen C., Tay Y. F., Chen S., Tang J., Wong L. (2022). Emerging Chalcogenide
Thin Films
for Solar Energy Harvesting Devices. Chem. Rev..

[2] Shin D., Saparov B., Mitzi D. B. (2017). Defect
Engineering in Multinary Earth-Abundant
Chalcogenide Photovoltaic Materials. Adv. Energy
Mater..

[3] Liang F., Kang L., Lin Z., Wu Y. (2017). Mid-Infrared
Nonlinear
Optical Materials Based on Metal Chalcogenides: Structure-Property
Relationship. Cryst. Growth Des..

[4] Chung I., Kanatzidis M. G. (2014). Metal Chalcogenides:
A Rich Source of Nonlinear Optical
Materials. Chem. Mater..

[5] Chen H., Wei W.-B., Lin H., Wu X.-T. (2021). Transition-metal-based
chalcogenides: A rich source of infrared nonlinear optical materials. Coord. Chem. Rev..

[6] Fernandes R. M., Coldea A. I., Ding H., Fisher I. R., Hirschfeld P. J., Kotliar G. (2022). Iron pnictides and chalcogenides:
a new paradigm for
superconductivity. Nature.

[7] Glasbrenner J. K., Mazin I. I., Jeschke H. O., Hirschfeld P. J., Fernandes R. M., Valentí R. (2015). Effect of
magnetic frustration on
nematicity and superconductivity in iron chalcogenides. Nat. Phys..

[8] Shi Y., Sturm C., Kleinke H. (2019). Chalcogenides as thermoelectric materials. J. Solid State Chem..

[9] Wei T.-R., Qiu P., Zhao K., Shi X., Chen L. (2023). Ag_2_Q-Based
(Q = S, Se, Te) Silver Chalcogenide Thermoelectric Materials. Adv. Mater..

[10] Han C., Sun Q., Li Z., Dou S. X. (2016). Thermoelectric Enhancement of Different
Kinds of Metal Chalcogenides. Adv. Energy Mater..

[11] Sato T., Segawa K., Guo H., Sugawara K., Souma S., Takahashi T., Ando Y. (2010). Direct Evidence for
the Dirac-Cone
Topological Surface States in the Ternary Chalcogenide TlBiSe_2_. Phys. Rev. Lett..

[12] Yi H., Hu L. H., Zhao Y. F. (2023). Dirac-fermion-assisted
interfacial superconductivity in epitaxial topological-insulator/iron-chalcogenide
heterostructures. Nat. Commun..

[13] Jana S., O’Donnell S., Leahy I. A., Koldemir A., Pöttgen R., Smaha R. W., Maggard P. A. (2024). Synthesis, crystal
structure, and
physical properties of the Eu­(II)-based selenide semiconductor: EuHfSe_3_. J. Mater. Chem. C.

[14] Jana S., Gabilondo E. A., Mongkhonratanachai M., Zhang Y., Halasyamani P. S., Maggard P. A. (2024). Large Mid-Infrared Second-Harmonic Generation in Eu­(II)-Based
Quaternary Chalcogenides. Chem. Mater..

[15] Panigrahi G., Morrison G., Smith M. D., zur Loye H.-C. (2024). Synthesis, Optical,
and Magnetic Properties of the Mixed Chalcogenide Semiconductor Series
Eu­(II)_2_SiSe_x_S_4-x_, Prepared via the
Flux-Assisted Boron Chalcogen Mixture Method. Inorg. Chem..

[16] Bruker APEX4; Bruker AXS Inc.: Madison, WI, 2012.

[17] Sheldrick, G. M. SADABS; University of Göttingen: Germany, 1996.

[18] Bruker XPREP; Bruker AXS Inc.: Madison, WI, 2012.

[19] Sheldrick G. M. (2015). *SHELXT* – Integrated space-group and crystal-structure
determination. Acta Crystallogr., Sect. A:Found.
Adv..

[20] Sheldrick G. M. (2015). Crystal
structure refinement with SHELXL. Acta Crystallogr.,
Sect. C:Struct. Chem..

[21] Spek A. L. (2003). Single-crystal
structure validation with the program PLATON. J. Appl. Crystallogr..

[22] Gelato L. M., Parthé E. (1987). *STRUCTURE TIDY* –
a computer
program to standardize crystal structure data. J. Appl. Crystallogr..

[23] Altomare A., Cuocci C., Giacovazzo C., Moliterni A., Rizzi R., Corriero N., Falcicchio A. (2013). EXPO2013:
a kit of tools for phasing crystal structures from powder data. J. Appl. Crystallogr..

[24] Toby B. H., Von Dreele R. B. (2013). GSAS-II: the genesis of a modern
open-source all purpose
crystallography software package. J. Appl. Crystallogr..

[25] Kurtz S. K., Perry T. T. (1968). A Powder Technique
for the Evaluation of Nonlinear
Optical Materials. J. Appl. Phys..

[26] Kortüm, G. ”Reflectance Spectroscopy: Principles, Methods, Applications”; Springer, 2012.

[27] Makuła P., Pacia M., Macyk W. (2018). How To Correctly Determine the Band
Gap Energy of Modified Semiconductor Photocatalysts Based on UV-Vis
Spectra. J. Phys. Chem. Lett..

[29] Nesper R. (2014). The Zintl-Klemm
Concept - A Historical Survey. Z. Anorg. Allg.
Chem..

[30] Zhou W., Guo S.-P. (2024). Rational Design of Novel Promising Infrared Nonlinear
Optical Materials: Structural Chemistry and Balanced Performances. Acc. Chem. Res..

[31] Abudurusuli A., Wu K., Pan S. (2018). Four new quaternary
chalcogenides A_2_Ba_7_Sn_4_Q_16_ (A = Li, Na; Q = S, Se): syntheses,
crystal structures determination, nonlinear optical performances investigation. New J. Chem..

[32] Zhen N., Wu K., Wang Y., Li Q., Gao W., Hou D., Yang Z., Jiang H., Dong Y., Pan S. (2016). BaCdSnS_4_ and Ba_3_CdSn_2_S_8_: syntheses,
structures, and non-linear optical and photoluminescence properties. Dalton Trans..

[33] Yang Y., Song M., Zhang J., Gao L., Wu X., Wu K. (2020). Coordinated regulation on critical physiochemical performances activated
from mixed tetrahedral anionic ligands in new series of Sr_6_A_4_M_4_S_16_ (A = Ag, Cu; M = Ge, Sn)
nonlinear optical materials. Dalton Trans..

[34] Feng P., Zhang J.-X., Ran M.-Y., Wu X.-T., Lin H., Zhu Q.-L. (2024). Rare-earth-based
chalcogenides and their derivatives:
an encouraging IR nonlinear optical material candidate. Chem. Sci..

[35] Wu K., Yang Z., Pan S. (2016). Na_2_Hg_3_M_2_S_8_ (M = Si, Ge, and Sn): New Infrared Nonlinear
Optical Materials with Strong Second Harmonic Generation Effects and
High Laser-Damage Thresholds. Chem. Mater..

[36] Griessen R., Landolt M., Ott H. (1971). A new antiferromagnetic
phase in
EuSe below 1.8 K. Solid State Commun..

[37] Wachter, P. Europium chalcogenides: EuO, EuS, EuSe and EuTe. In Handbook on the Physics and Chemistry of Rare Earths; Elsevier, 1979, Chapter 19, pp 507–574.

[38] Li D. X., Yamamura T., Nimori S., Homma Y., Honda F., Aoki D. (2013). Giant and isotropic
low temperature magnetocaloric effect in magnetic
semiconductor EuSe. Appl. Phys. Lett..

