# Supplementary material for: Magnetic Properties and Large Second-Harmonic Generation Response of a Chiral Ternary Chalcogenide: Eu2SiSe4
Source: Chem Mater. 2025 Jul 9;37(14):5036–42. doi: 10.1021/acs.chemmater.5c00421 (PMC12287995; doi:10.1021/acs.chemmater.5c00421)
Supplement: Supplementary file 1 [file cm5c00421_si_001.pdf]

Supporting Information

Magnetic Properties and Large Second-Harmonic Generation Response of a Chiral Ternary Chalcogenide: Eu_2SiSe_4

Shaun O'Donnell,[†,||] Ian A. Leahy,[†,||] Subhendu Jana,[‡] Eric A. Gabilondo,[¶] P. Shiv Halasyamani,[¶] Paul A. Maggard,[*,‡] and Rebecca W. Smaha[*,§]

†*Materials Science Center, National Renewable Energy Laboratory, Golden, CO 80401, USA*

‡*Department of Chemistry and Biochemistry, Baylor University, Waco, TX 76798, USA*

¶*Department of Chemistry, University of Houston, Houston, TX 77204, USA*

§*Materials Science Center, National Renewable Energy Laboratory, Golden, Colorado 80401, USA*

||*Equal Contribution*

E-mail: Paul_Maggard@baylor.edu; Rebecca.Smaha@nrel.gov

Contents

Crystallographic Details **S1**

Powder X-ray Diffraction Data **S4**

Additional SHG Data **S6**

Additional Magnetic Data **S7**

Additional Thermodynamic Data **S11**

References **S14**

Crystallographic Details

The Si1–Se distances in the 300 K Eu_2SiSe_4 crystal structure of 2.255(1)–2.264(2) Å as listed in Table S4 can be compared with the previously reported compounds such as $Ba_4SiSb_2Se_{11}$ (2.270(3)-–2.297(3) Å)[1] and $Na_2EuSiSe_4$ (2.246(2)–2.288(2) Å).[2] The smallest Eu···Eu distance (Eu1···Eu2) in the Eu_2SiSe_4 crystal structure is 4.1839(4) Å and 4.1626(5) Å at 300 K and 100 K, respectively.

Table S1: Selected SCXRD refinement details of Eu_2SiSe_4 measured with λ = 0.71073 Å

Temperature (K)	300(2)	100(2)
Space group	$P2_1$	$P2_1$
a (Å)	6.8270(4)	6.8093(7)
b (Å)	6.9535(4)	6.9660(7)
c (Å)	8.4356(4)	8.3828(7)
β (°)	108.141(2)	108.208(3)
V (Å^3)	380.55(4)	377.72(6)
ρ (g cm^{-3})	5.654	5.696
Z	2	2
μ (mm^{-1})	35.50	35.77
$R(F)^a$	0.019	0.020
$R_w(F_o^2)^b$	0.043	0.041
S	1.06	1.00
No. of measured reflections	14108	3614
No. of independent reflections	2295	1773
R_{int}	0.057	0.031
Flack x	0.80(2)	0.24(3)
$\Delta\rho_{max}$, $\Delta\rho_{min}$ (eÅ^{-3})	1.03, -1.26	1.09, -1.14

^{a} $R(F) = \Sigma||F_o| - |F_c||/\Sigma|F_o|$ for $F_o^2 > 2\sigma(F_o^2)$

^{b} $R_w(F_o^2) = \Sigma[w(F_o^2 - F_c^2)^2]/\Sigma w F_o^{4\,1/2}$. For $F_o^2 < 0$, $w = 1/[\sigma(F_o^2) + (mP)^2 + nP]$, where $P = (F_o^2 + 2F_c^2)/3$. m and n values are 0.0123 and 0.0993 for 300 K data, 0, 0 for 100 K data, respectively.

Table S2: Atomic positions, bond valence sum (BVS), site symmetry, Wyckoff positions, and equivalent isotropic displacement parameters in the Eu_2SiSe_4 crystal structure derived from SCXRD at 300 K. All atom sites are fully occupied.

Atom	BVS	Wyckoff Position	Site Symmetry	*x*	*y*	*z*	*U*eq
Eu1	1.82	2*a*	1	0.22010(4)	0.53984(4)	0.55157(3)	0.01713(8)
Eu2	1.73	2*a*	1	0.26677(4)	0.54400(5)	0.06867(3)	0.01864(9)
Si1	4.01	2*a*	1	0.2222(2)	0.0209(3)	0.29905(15)	0.0119(3)
Se1	2.02	2*a*	1	0.00993(9)	0.27792(8)	0.76539(9)	0.01341(13)
Se2	2.03	2*a*	1	0.01602(10)	0.28252(8)	0.24255(8)	0.01478(13)
Se3	1.69	2*a*	1	0.39452(9)	0.00007(11)	0.57396(7)	0.02113(16)
Se4	1.82	2*a*	1	0.41606(9)	0.00711(13)	0.12577(7)	0.02195(16)

Table S3: Atomic positions, Bond valence sum (BVS), site symmetry, Wyckoff positions, and equivalent isotropic displacement parameters in the Eu_2SiSe_4 crystal structure derived from SCXRD at 100 K. All atom sites are fully occupied.

Atom	BVS	Wyckoff Position	Site Symmetry	*x*	*y*	*z*	*U*eq
Eu1	1.88	2*a*	1	0.21973(6)	0.54267(6)	0.55190(4)	0.00679(11)
Eu2	1.78	2*a*	1	0.26493(6)	0.54969(6)	0.06807(4)	0.00716(11)
Si1	3.98	2*a*	1	0.2231(3)	0.0160(4)	0.3000(2)	0.0062(5)
Se1	2.03	2*a*	1	0.01579(13)	0.27726(12)	0.76660(11)	0.00586(18)
Se2	2.07	2*a*	1	0.02222(13)	0.28092(12)	0.24480(11)	0.00616(18)
Se3	1.72	2*a*	1	0.39779(13)	-0.01457(13)	0.57559(8)	0.00812(18)
Se4	1.82	2*a*	1	0.41661(12)	-0.00478(13)	0.12467(8)	0.00846(19)

Table S4: Inter-atomic distances in the Eu_2SiSe_4 crystal structure at 300 K and 100 K, respectively.

Atom pair	Distance (Å) at 300 K	Distance (Å) at 100 K
Si1—Se1	2.2637(18)	2.271(3)
Si1—Se2	2.258(2)	2.257(3)
Si1—Se3	2.2548 (14)	2.2537(19)
Si1—Se4	2.2591(14)	2.265(2)
Eu1—Se1	3.2001(7)	3.1856(9)
Eu1—Se1	3.1211(7)	3.1137(10)
Eu1—Se2	3.1107(7)	3.0979(9)
Eu1—Se2	3.1946(7)	3.1824(10)
Eu1—Se3	3.3992(8)	3.2973(10)
Eu1—Se3	3.1393(7)	3.1342(9)
Eu1—Se4	3.0692(6)	3.0653(8)
Eu2—Se1	3.2015(7)	3.1916(10)
Eu2—Se1	3.1338(7)	3.1214(9)
Eu2—Se2	3.1960(7)	3.1767(9)
Eu2—Se2	3.1546(8)	3.1562(10)
Eu2—Se3	3.1918(6)	3.1797(8)
Eu2—Se4	3.1075(6)	3.1059(8)
Eu2—Se4	3.3667(10)	3.2585(10)
Eu1—Eu1	4.4998(4)	4.4962(6)
Eu1—Eu2	4.2737(4)	4.2412(5)
Eu1—Eu2	4.1839(4)	4.1626(5)
Eu1—Si1	3.6217(14)	3.609(2)
Eu1—Si1	3.6180(14)	3.607(2)

Powder X-ray Diffraction Data

The PXRD data of the solid-state (SS) and BCM samples on which SHG, magnetic, and thermodynamic measurements were performed are compared in Fig. S1. Figure S2 magnifies the region near the most intense peak from the EuSe impurity phase. We performed Rietveld refinements upon each dataset to extract the phase fractions. The SS sample exhibited 14.7(2) wt.% EuSe while the BCM sample contained only 2.5(2) wt.% EuSe. We also performed a Rietveld refinement of a second solid-state sample (SS-2) upon which SHG activity measurements were also performed (see Fig. S3); this sample also contained approximately 15 wt.% EuSe.

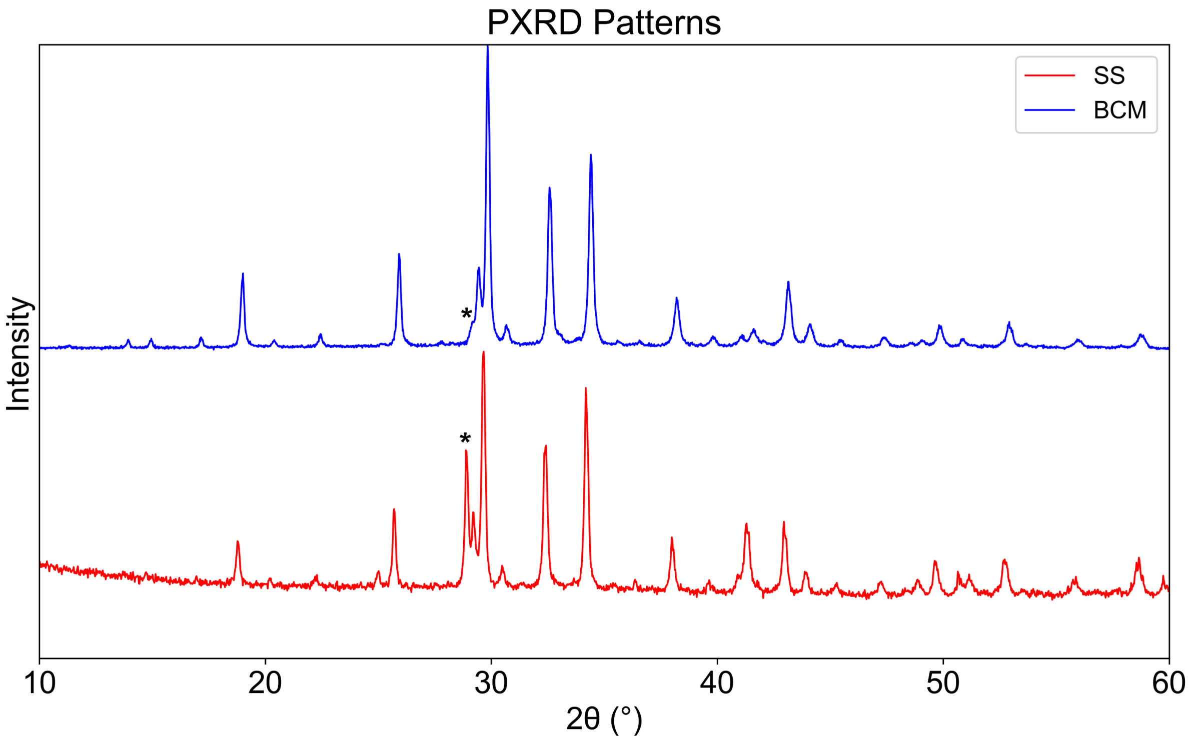

Figure S1: Comparison of powder x-ray diffraction data of Eu_2SiSe_4 synthesized via solid-state (SS) and BCM methods. The asterisk indicates the most intense peak from the EuSe impurity (s.g. 225).

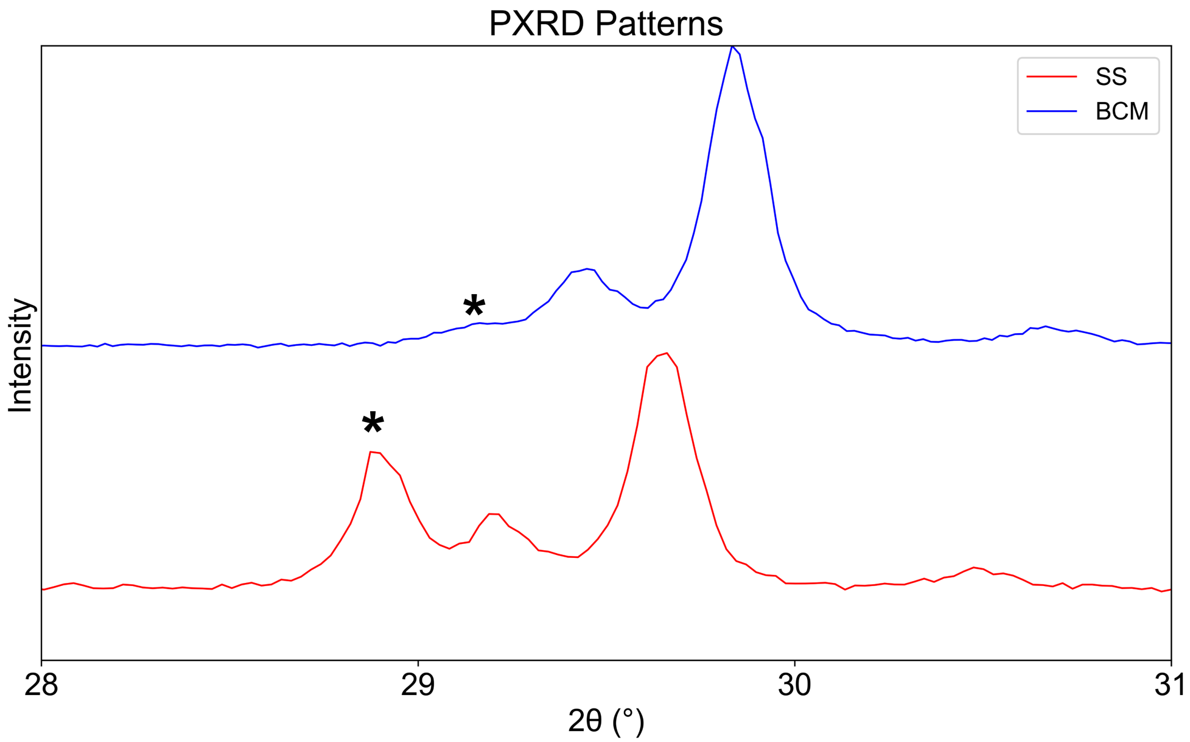

Figure S2: Magnified comparison of powder x-ray diffraction data of Eu_2SiSe_4 synthesized via solid-state (SS) and BCM methods. The asterisk indicates the most intense peak from the EuSe impurity. The apparent shift in peak position is likely due to height difference between the samples.

Additional SHG Data

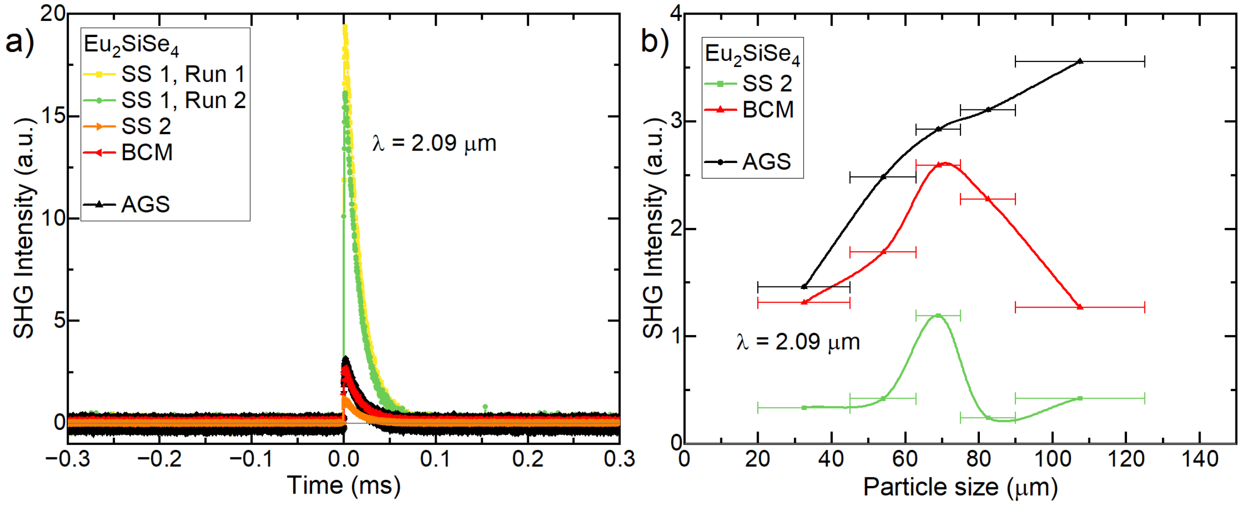

Figure S3: a) SHG response at 2.09 μm of Eu_2SiSe_4 against AGS ($AgGaS_2$), comparing two samples synthesized via the solid-state method (SS 1, SS2) and one synthesized via the BCM method. The SS 1 sample was measured twice to check the consistency. The particle sizes of the SS, BCM, and AGS samples were ∼1–50, 63–75, and 63–75 μm, respectively. b) Phase matching curve for a second solid-state sample (SS 2) and the BCM sample against AGS at 2.09 μm.

Additional Magnetic Data

Magnetic measurements were additionally performed on the Eu_2SiSe_4 sample synthesized using the two-step solid-state method that contained 14.7(2) wt.% EuSe. Magnetic susceptibility as a function of temperature under a variety of applied magnetic fields is compared between the BCM and SS samples in Fig. S4. From this comparison, we can deduce that EuSe is the likely primary origin of the magnetic behavior at ∼4.7 K, as this feature is significantly enhanced in the solid-state sample.

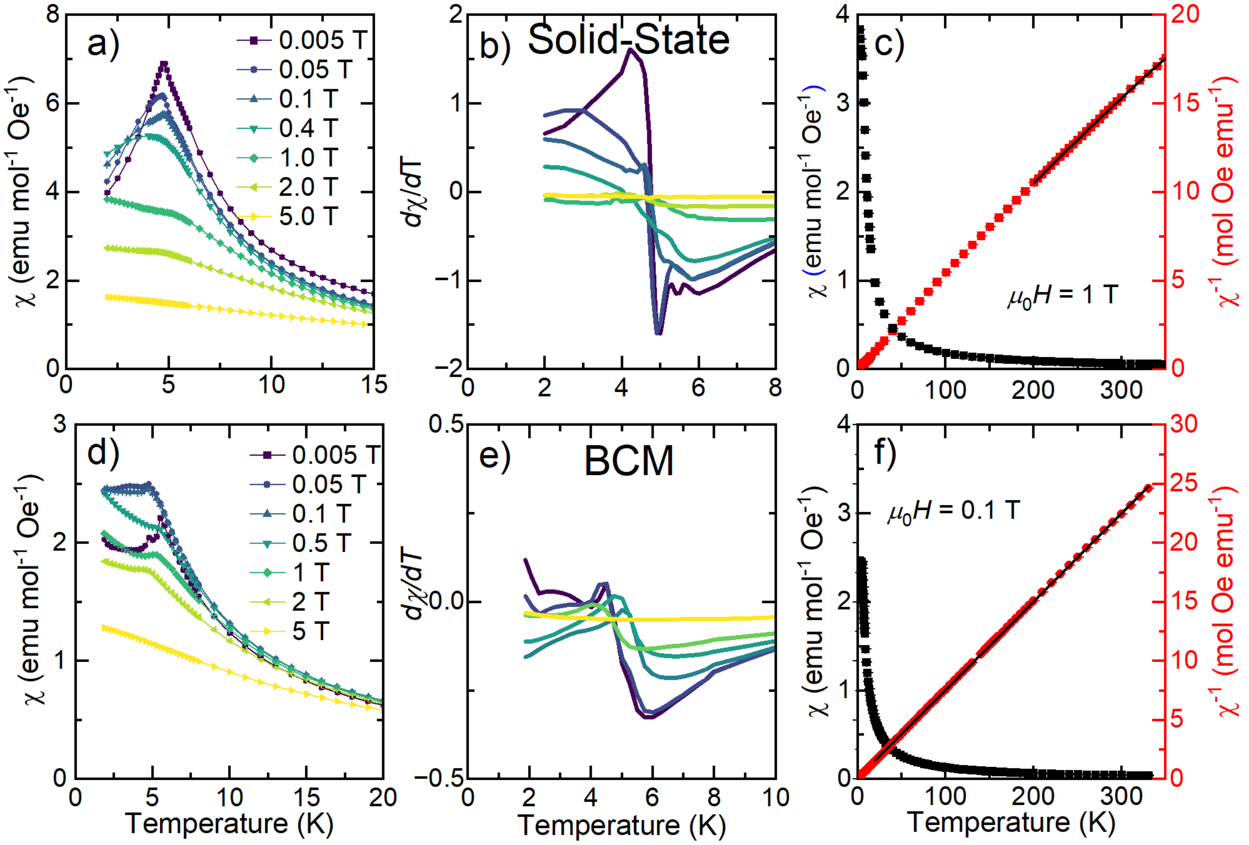

Figure S4: Comparison of magnetic susceptibility of Eu_2SiSe_4 synthesized via solid-state (top) and BCM (bottom) methods. (a,d) Low temperature magnetic susceptibility (χ) as a function of temperature measured at several applied fields. (b,e) Derivative of χ as a function of temperature. (c,f) Susceptibility and inverse susceptibility (χ^{-1}) measured in an applied field of $\mu_0 H = 1$ T. The Curie-Weiss fit is the black line. All data are zero-field cooled (ZFC) unless otherwise stated in the legend.

We performed Curie-Weiss fits on high temperature inverse susceptibility data from both samples, as shown in Fig. S4c,f. The fit of the BCM sample is described in the main text. The fit of the SS sample was performed from 200 – 350 K with diamagnetic correction $\chi_0 = 9(1) \times 10^{-3}$ emu/mol, as shown in Fig. S4C. This yielded a Curie constant $C = 16.4(5)$ K·emu/mol per formula unit and an effective moment of 8.1(1) μ_B per Eu, consistent with the 2+ oxidation state suggested by BVS calculations. This suggests that the SS method is less likely to oxidize the sample than the BCM method, likely due to the absence of a washing step. The Weiss temperature $\Theta = 11(4)$

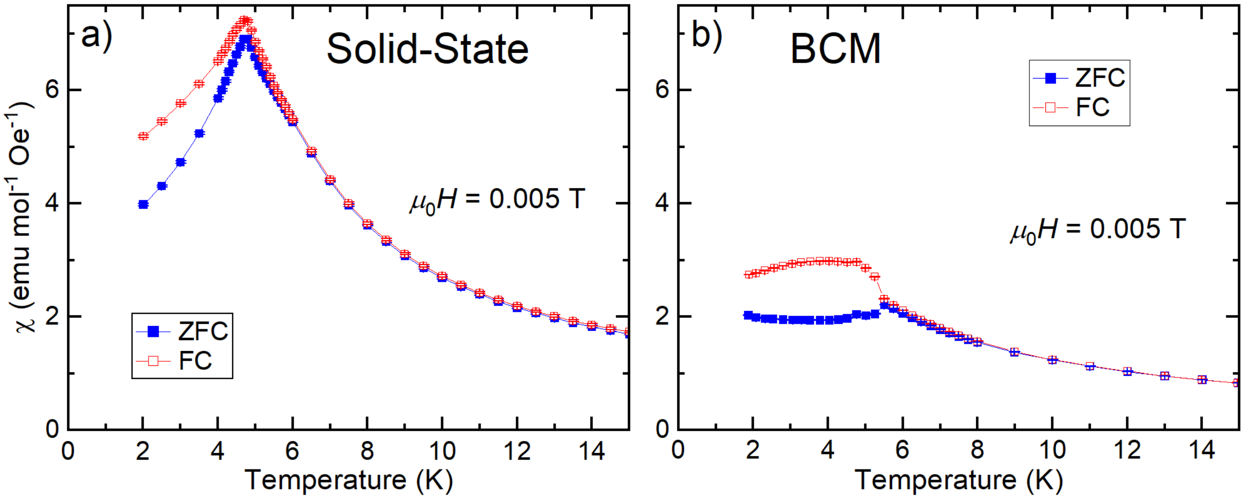

Figure S5: Zero-field cooled (ZFC) and field cooled (FC) magnetic susceptibility of Eu_2SiSe_4 synthesized by both a) solid-state and b) BCM methods.

K of the SS sample is consistent with weak ferromagnetic interactions, as compared to the weak antiferromagnetic Θ extracted from the BCM sample. Both samples exhibit splitting between the zero-field-cooled and field-cooled data at low applied field, as shown in Fig. S5; this is consistent with antiferromagnetic ground states and may indicate some glassy character.

We note that the susceptibility data of the SS sample also revealed a small transition at approximately $T_1^* = 145$ K, shown in Fig. S6, that is slightly suppressed at high field. Because this transition is not seen in the BCM sample, we postulate that it likely arises from the EuSe impurity that is present in a larger amount in the SS sample.

Magnetization as a function of applied field was measured at several temperatures for both samples, compared in Fig. S7. The data for the SS sample is more complex below the ordering temperature of EuSe (4.7 K) than the BCM sample data, consistent with the larger amount of EuSe present. Interestingly, a tiny net moment is present at 2 K in the SS sample but not in the BCM sample, consistent with the ferrimagnetic ground state of EuSe[3–6] and with the small positive Weiss temperature observed for this sample. The derivative of magnetization as a function of applied field (Fig. S7e,f) exhibits multiple peaks at low field for temperatures below 6 K (see insets), consistent with the multiple slopes observed in the magnetization at 2 K and the multiple phases present. The peaks in dM/dH shift towards lower applied fields as the temperature increases, indicative of the weakening of the magnetic interactions at higher temperatures. By comparing the two datasets, it becomes apparent that the strong features at ∼3 T and ∼0.5 T arise primarily from EuSe.

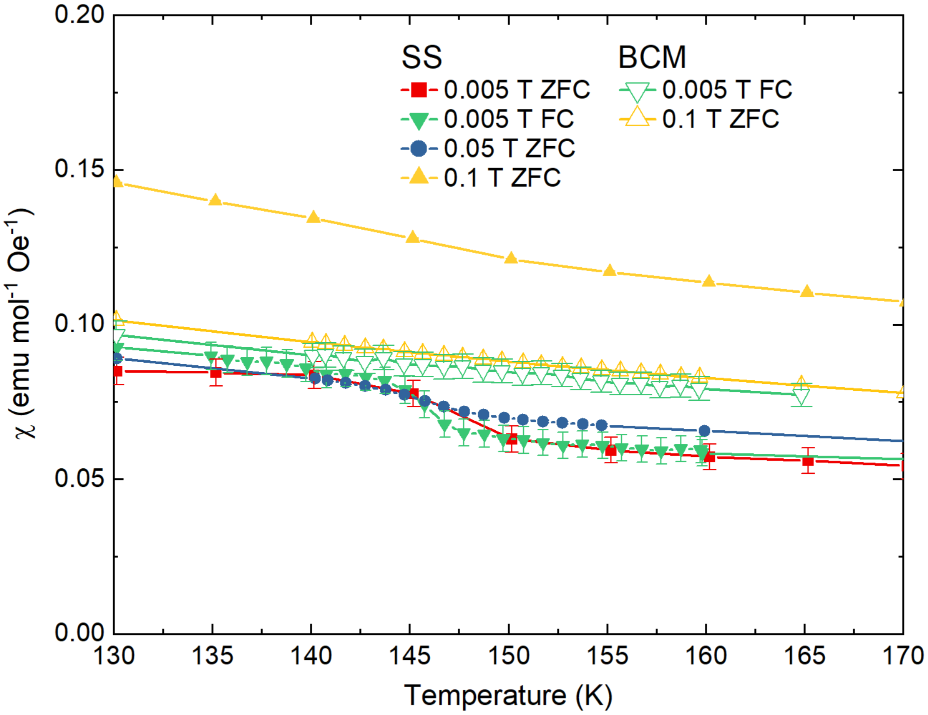

Figure S6: High temperature magnetic susceptibility comparing the solid-state (SS) and BCM samples.

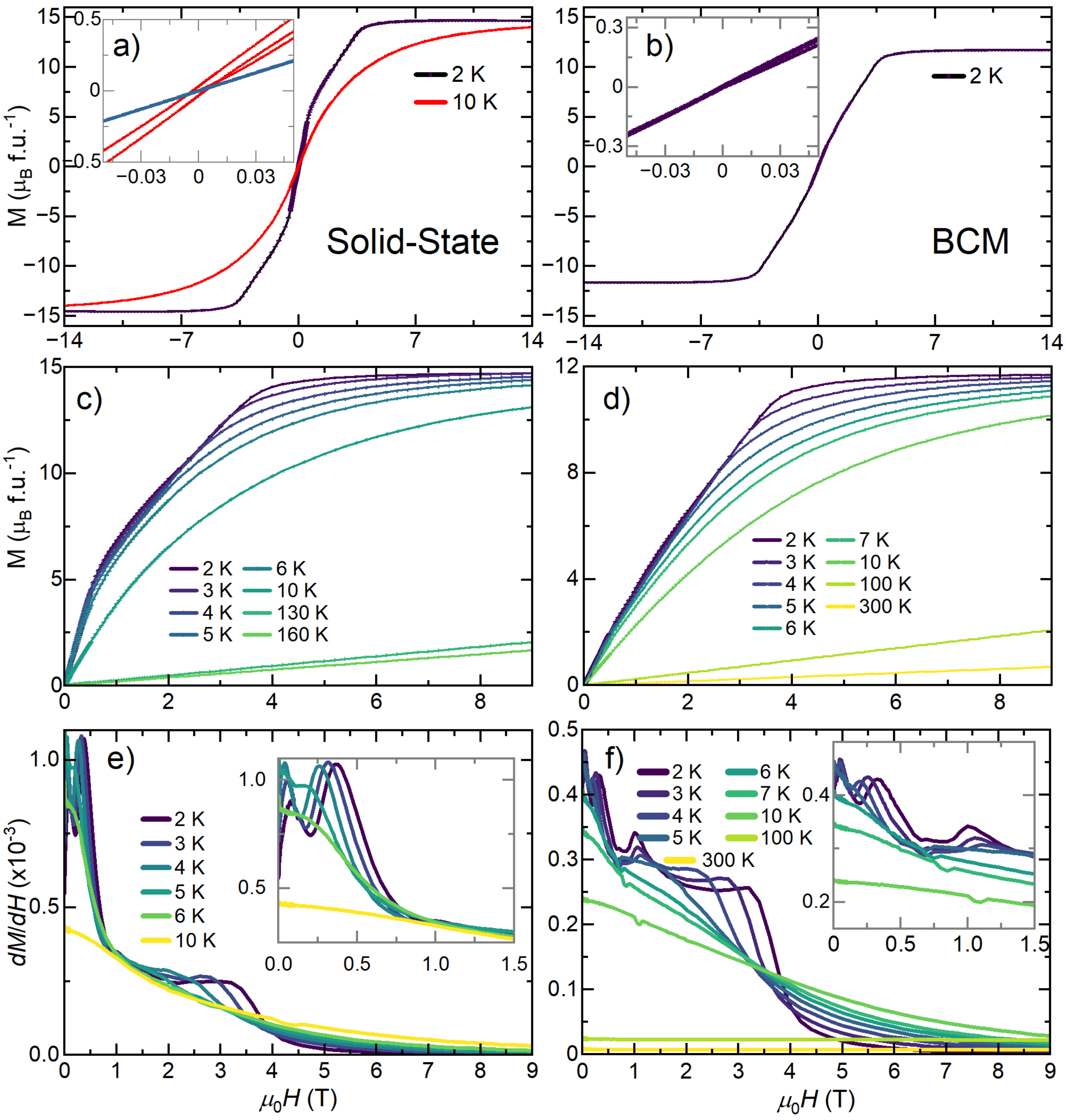

Figure S7: Comparison of magnetization of Eu_2SiSe_4 synthesized via solid-state (left) and BCM (right) methods. (a,b) DC magnetization as a function of applied field at low temperature. Inset magnifies the data near zero field. (c,d) DC magnetization as a function of applied field at a range of temperatures. (e,f) Derivative of magnetization as a function of applied field at several temperatures. Inset magnifies the data at low field.

Additional Thermodynamic Data

Figure S8 compares the heat capacity of Eu_2SiSe_4 synthesized via solid-state (left) and BCM (right) methods from 1.8 - 30 K at fixed magnetic fields up to 14 T. In the SS sample data, at 0 T two peaks are clearly visible, consistent with its susceptibility. With increasing field, the lower of these peaks is quickly wiped away, while the T_N peak moves to lower temperatures and broadens. The peak at 4.7 arises from EuSe.

To analyze the magnetic contributions to the heat capacity, we fit the 0 T heat capacity for both samples above 30 K to Equation 1, a Debye model with a two-level Schottky contribution to account for thermally activated crystal electric field (CEF) split states (black dashed lines in Fig. S9). We anticipate that the disagreement of the experimental S_{Mag} originates from CEF effects and the effect of the EuSe impurity phase. This is reflected in the magnetic field dependence of the heat capacity far above T_N as well as the broadness of this dependence in temperature. It is likely the case that the manifold of CEF states is not faithfully captured by our simple two-level model. In future work, we anticipate that a more complete understanding of energy splittings between the most relevant Eu^{2+} CEF states would resolve this discrepancy.

Figure S10 shows the heat capacity from 120 – 175 K highlighting the slight transition observed in the solid-state sample but less obvious in the BCM sample. We therefore tentatively attribute this transition to EuSe.

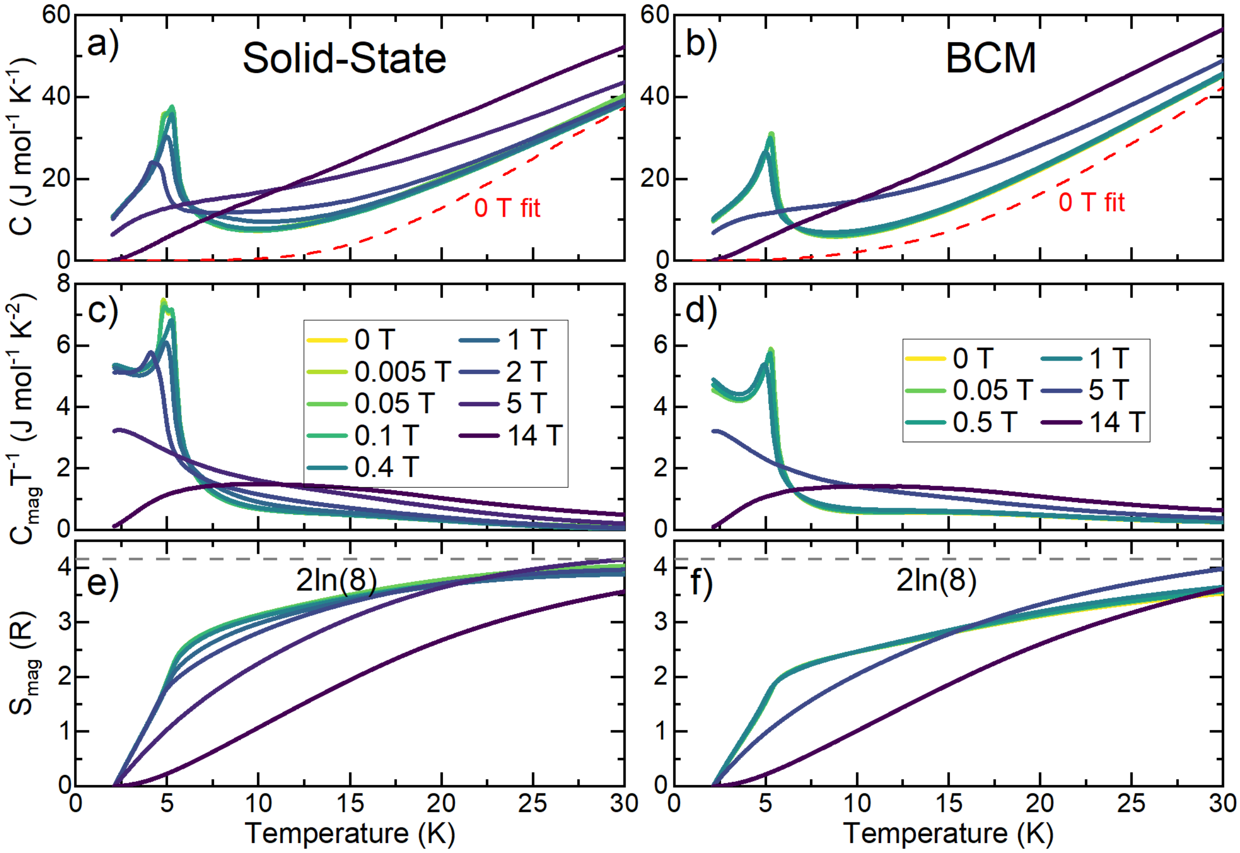

Figure S8: Heat capacity for polycrystalline Eu_2SiSe_4 synthesized by both solid-state (SS, left) and BCM (right) methods.
(a,b) Molar heat capacity (C_p) at several applied fields; black dashed line represents the background (C_{bg}) for the $\mu_0 H = 0$ T curve, which was fit above 30 K to Eq. 1 and extrapolated to zero. (c,d) Heat capacity related to the low temperature peaks (C_{mag}). (e,f) Magnetic entropy at low temperature (S_{mag}) normalized per formula unit.

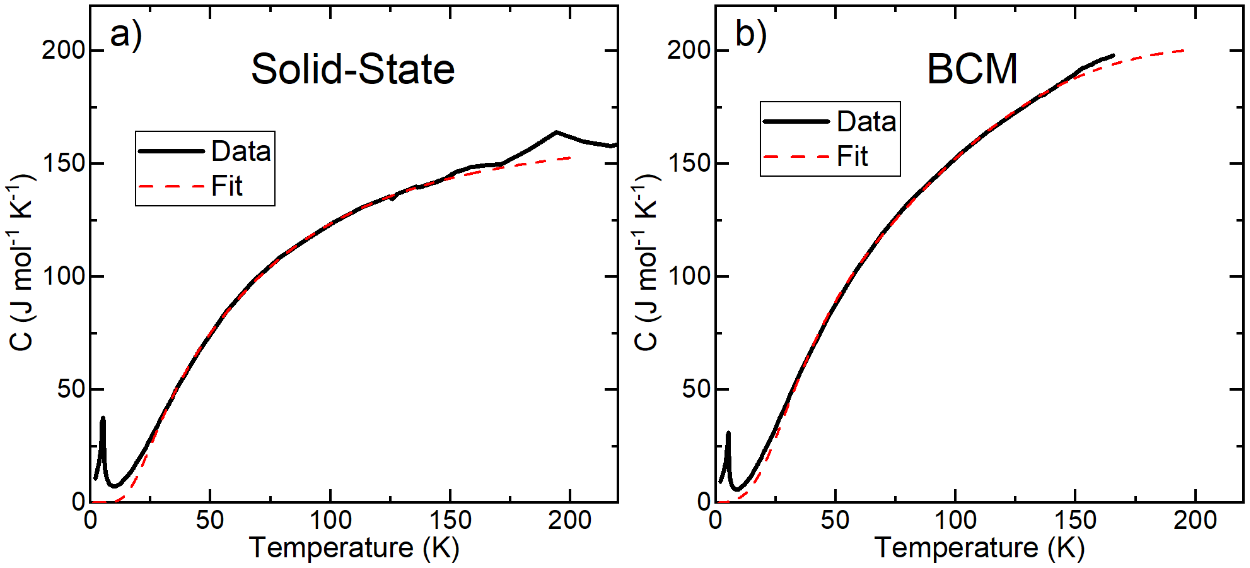

Figure S9: Background fits of heat capacity data collected at $\mu_0 H = 0$ T of the Eu_2SiSe_4 samples synthesized via the a) solid-state and b) BCM methods.

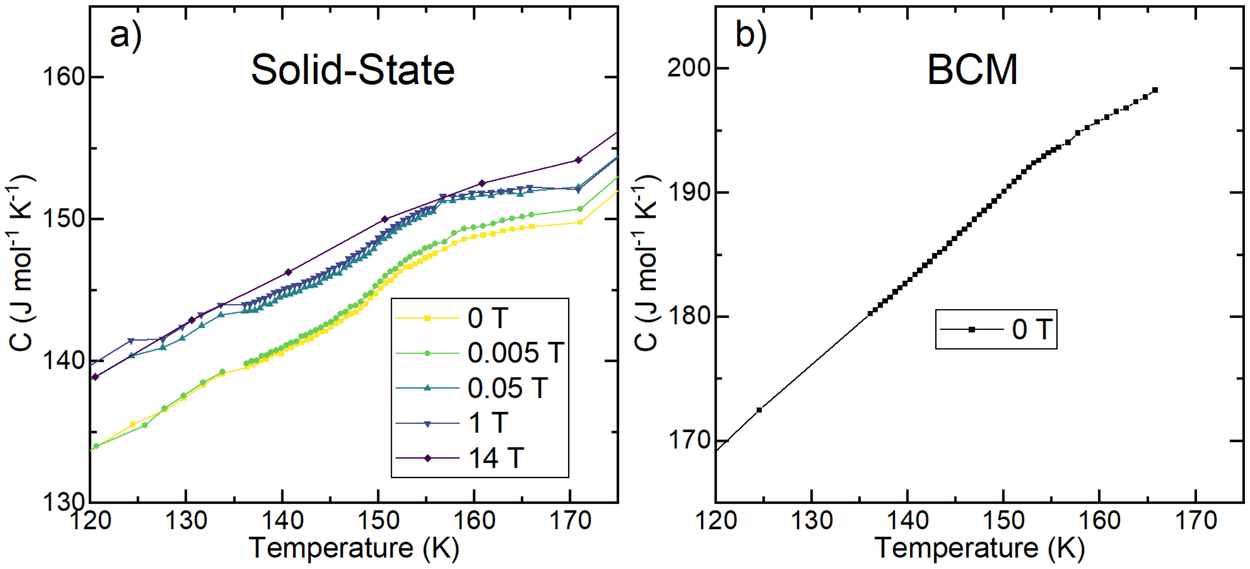

Figure S10: Heat capacity highlighting the $T_1^* \approx 145$ K transition observed in the a) solid-state sample but less obvious in the b) BCM sample.

References

(1) Choi, K.-S.; Kanatzidis, M. G. Si Extraction from Silica in a Basic Polychalcogenide Flux. Stabilization of Ba4SiSb2Se11, a Novel Mixed Selenosilicate/Selenoantimonate with a Polar Structure. *Inorganic Chemistry* **2001**, *40*, 101–104, PMID: 11195365.

(2) Choudhury, A.; Dorhout, P. K. An Ordered Assembly of Filled Nanoscale Tubules of Europium Seleno-silicate in the Crystal Structure of a Quaternary Compound. *Journal of the American Chemical Society* **2007**, *129*, 9270–9271, PMID: 17625863.

(3) Griessen, R.; Landolt, M.; Ott, H. A new antiferromagnetic phase in EuSe below 1.8 K. *Solid State Communications* **1971**, *9*, 2219–2223.

(4) Wachter, P. *Handbook on the Physics and Chemistry of Rare Earths*; Elsevier, 1979; Chapter EUROPIUM CHALCOGENIDES: EuO, EuS, EuSe AND EuTe.

(5) Li, D. X.; Yamamura, T.; Nimori, S.; Homma, Y.; Honda, F.; Aoki, D. Giant and isotropic low temperature magnetocaloric effect in magnetic semiconductor EuSe. *Applied Physics Letters* **2013**, *102*, 152409.

(6) Patiño, J. C.; Neto, D. P.; Gomes, A. M. Low-Temperature Magnetization, Calorimetric Properties, and Magnetocaloric Effect of Europium Chalcogenides EuSe and $EuSe_{0.90}S_{0.10}$. *IEEE Transactions on Magnetics* **2024**, *60*, 2500805.
